# Electron Transfer Rates in Polar and Non-Polar Environments: a Generalization of Marcus’ Theory to Include an Effective Treatment of Tunneling Effects

**DOI:** 10.1021/acs.jpclett.2c02343

**Published:** 2022-09-27

**Authors:** Anna Leo, Andrea Peluso

**Affiliations:** Dipartimento di Chimica e Biologia Università di Salerno, I-84084 Fisciano, Salerno Italy

## Abstract

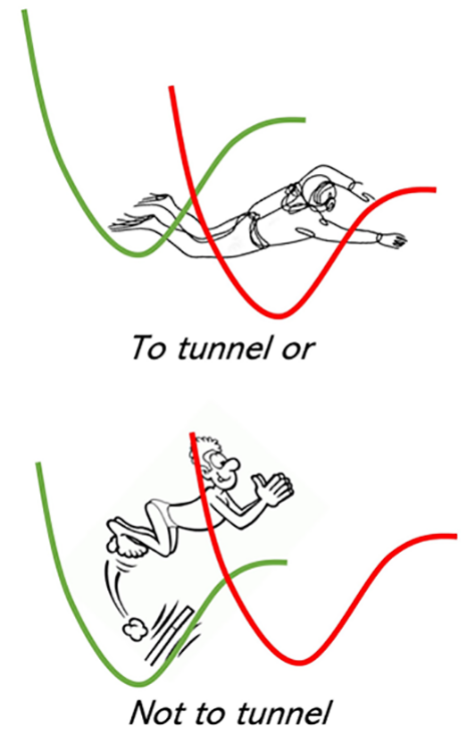

A multistep kinetic model in which solvent motion is treated in the framework of Marcus theory and the rates of the elementary electron transfer step are evaluated at full quantum mechanical level is proposed and applied to the calculation of the rates of intramolecular electron transfer reactions in rigidly spaced D–Br–A (D = 1,1′-biphenyl radical anion, Br = androstane) compounds, for five acceptors (A) in three organic solvents with different polarity. The calculated rates agree well with experimental ones, and their temperature dependence is almost quantitatively reproduced.

Electron transfer (ET) reactions are ubiquitous in chemistry and play a relevant role in modern technological applications such as solar cells, molecular electronic devices, biosensing, and photocatalysis. Marcus’ theory is the cornerstone for understanding mechanistic aspects of ET reactions and still represents the most robust theoretical framework for the rationale design of new chromophores to employ in technological devices.^1−3^ Indeed, it relates the kinetics of the ET process to physically sound quantities: the Δ*G*^0^ of the whole ET reaction and the reorganization energy associated with the motions of both solvent and reactants. However, because of the classical treatment of nuclear motion, Marcus’ rate expression is often unable to reproduce the observed temperature dependence of ET rates, with valuable exceptions where, however, the temperature dependence of the reorganization energy and the reaction free energy is accounted for,^4^ posing problems for a deeper understanding of important mechanistic aspects of several phenomena, as for instance charge transport in organic semiconductors, where tunneling likely plays an important role. Further problems have been found for strongly exothermic ET processes, for which predicted rates are many orders of magnitude slower than the observed ones.^5^ There are several outstanding works which have extended Marcus’ theory to include quantum effects due to high frequency modes,^6−13^ but, to the best of our knowledge, the task of including the whole heat bath provided by intramolecular coordinates of the redox pair, which was revealed to be crucial for reproducing the unusual temperature dependence of ET from bacteriopheophytin anions to primary ubiquinone in bacterial reaction centers,^14−16^ has not been undertaken yet for systems in which polar solvents are expected to play a significant role.

With that purpose in mind, herein, we generalize a recently proposed multistep kinetic model of ET reactions,^17,18^ which by separating the motion of the solvent from the internal one of the redox pair, makes it possible the use of a treatment of tunnelling effects which includes the whole set of nuclear coordinates of the redox pair and accounts for changes of both the equilibrium nuclear positions and vibrational frequencies upon electronic transition, as well as the effects due to normal mode mixing.^14,19,20^ The proposed model is then successfully applied to a set of electron transfer reactions in rigidly spaced donor-bridge-acceptor systems, for which ET rates and, in some cases, their temperature dependence have been experimentally determined in solvents of different polarity.^21,22^

The multistep mechanism is shown in Scheme 1, where D^–^A and DA^–^ denote the initial and the final states, respectively, and {D^–^A}* and {DA^–^}* denote the ensembles of transient structures in vibronic resonance with each other.

**Scheme 1:**
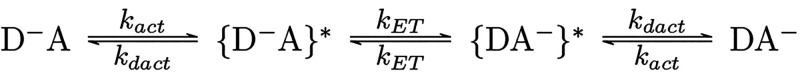
Multistep Kinetic Mechanism of ET

Step 1 is an activation step which brings the donor and the acceptor species into electronic degeneracy, thus triggering step 2, i.e., the elementary ET reaction. The backward reaction of step 1 and the forward one of step 3 account for relaxation of all of the nonequilibrium species to their minimum energy structures, including also the environment. The activation step is necessary in all of those case in which the initial and final states are not in vibronic resonance at frozen solvent coordinates, a situation which can often occur in polar environments, because the solvent polarization free energy can be larger than the Δ*G*^0^ of the ET reaction. For the activation step, we thus assume that only solvent coordinates are involved and that its rate constant has the usual Arrhenius dependence on temperature:
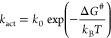
1where *k*_0_ is a transmission coefficient and Δ*G*^#^ is the standard activation free energy, i.e., the free energy difference of {D^–^A}* with respect to D^–^A, which, closely following Marcus’ reasoning, but at frozen intramolecular coordinates,^3^ is given by
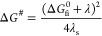
2where Δ*G*_fi_^0^ is the free energy difference between initial and final states, λ_s_ and λ_i_ are the reorganization energies of solvent and of the redox pair A and D, respectively, and λ = λ_s_ + λ_i_. Derivation of eq 2 is straightforward and is based on the same assumptions made by Marcus in his original work,^1,3^ but for the sake of clarity it is reported in the Supporting Information. It is noteworthy that Δ*G*^#^ is higher than in Marcus’ theory, because of our assumption that only solvent coordinates are involved in the activation step.

Backward step 1 and forward step 3 consist of the solvent response to a nonequilibrium charge distribution of the solute. Those processes have been extensively studied by time dependent spectroscopic measurements (Stokes shifts), and their rates have been experimentally determined.^23,24^ Experimental values from time dependent spectroscopic measurements will be used throughout for *k*_dact_. The latter quantity is related to *k*_0_ by the principle of microscopic reversibility, which must hold for the activation step, even though neither equilibrium nor steady state has been assumed, because of its purely mechanical origin, based on the invariance of the mechanical equations of motion under the operation of time reversal.^25,26^ The principle of microscopic reversibility implies the following relation between *k*_act_ and *k*_dact_, averaged over all of the possible paths bringing degenerate reactants to degenerate products:^25^
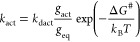
3where *g*_act_ and *g*_eq_ are the total degeneracies of the activated and the equilibrium states. The above condition implies:
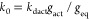
4Since the degeneracy factors *g*_i_ cannot be easily estimated, here we have left *k*_0_ as the only solvent dependent adjustable parameters to be fixed by comparison with experimental rates.

Step 2 is the elementary ET process under resonant conditions, whose rate can be reliably calculated by resorting to first order time dependent perturbation theory, i.e., the Fermi Golden Rule (FGR), according to which the rate of a nonradiative transition between two electronic states |*i*⟩ and |*f*⟩ is given by
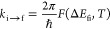
5where *F*(Δ*E*_fi_,*T*) is
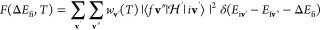
6where  is the electronic coupling element for the ET reaction, **v**′ and **v**″ denote the vectors of the vibrational quantum states of the |*i*⟩ and |*f*⟩, respectively, *E*_i**v**′_ and *E*_f**v**″_ are the vibronic energies of |*i***v**′⟩ and |*f***v**”⟩, respectively, and *w*_**v**′_ is the equilibrium (Boltzmann) population of |*i***v**′⟩.

By making the reasonable assumption that the dependence of the electronic coupling elements on vibrational coordinates can be neglected, eq 6 becomes
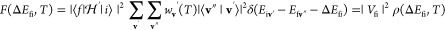
7where ρ(Δ*E*_fi_, *T*) is the Franck–Condon weighted density of states of the elementary |*i*⟩ → |*f*⟩ transition at the Δ*E* of the reaction, averaged over a thermal equilibrium distribution of initial vibrational states. When dealing with ET between two weakly interacting molecules, by taking advantage that vibrational motions of each molecular site upon electron transfer can be assumed to be independent from each other, the ρ(Δ*E*_fi_,*T*) for the ET process D^–^ + A → D + A^–^ can be obtained by the convolution of the ρ’s of the two redox half reactions D^–^ → e^–^ + D and A + e^–^ → A^–^:
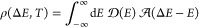
8In the equation above,  and  are the spectral distributions of the donor photoelectronic and of the acceptor electron attachment spectra.^27,28^ Both  and  are experimentally accessible quantities,^29−31^ and both can also be reliably obtained by first principle calculations.^14,28,32−34^ Indeed, upon the assumption that nuclear motion can be modeled in harmonic approximation, an explicit expression for *f*(λ), the Laplace transform of *F*(Δ*E*,*T*), can be obtained by introducing the density matrix of the harmonic oscillator in the coordinate representation and evaluating the trace by performing integration over nuclear coordinates.^19^ From *f*(λ), ρ(Δ*E*,*T*) can be obtained by using the inverse Laplace transformation.^14,19,20,28,32^ Very accurate estimates of ρ(Δ*E*,*T*) of both radiative and nonradiative transitions have been obtained in the past by using this procedure.^35−39^

The chemical structures of the donor and the acceptors considered here are drawn in Figure 1. The energies of the initial A D^–^ A and final DA^–^ states, computed at the density functional theory (DFT) level, using the continuum polarizable medium (PCM) approach for evaluating the contribution of solvent polarization, see Computational Details, are reported in Table 1, for the three solvents, iso-octane, tetrahydrofuran (THF), and dibutylether (DBE), for which experimental measurements are available.^21,40^ Calculated electronic energy differences for ET from donor to each acceptor agree well with the corresponding experimental ET free energies (Δ*G*^0^), as shown in Figure S1, where theoretical Δ*E*_fi_ are plotted against measured Δ*G*^0^’s. Hereafter, we will consider the computed Δ*E*_fi_ as free energies, assuming, as usual,^3^ that the entropy of reaction due to intramolecular vibrations is negligible. Entropy contributions of solvent motions are instead accounted for by λ_s_.

**Figure 1:**
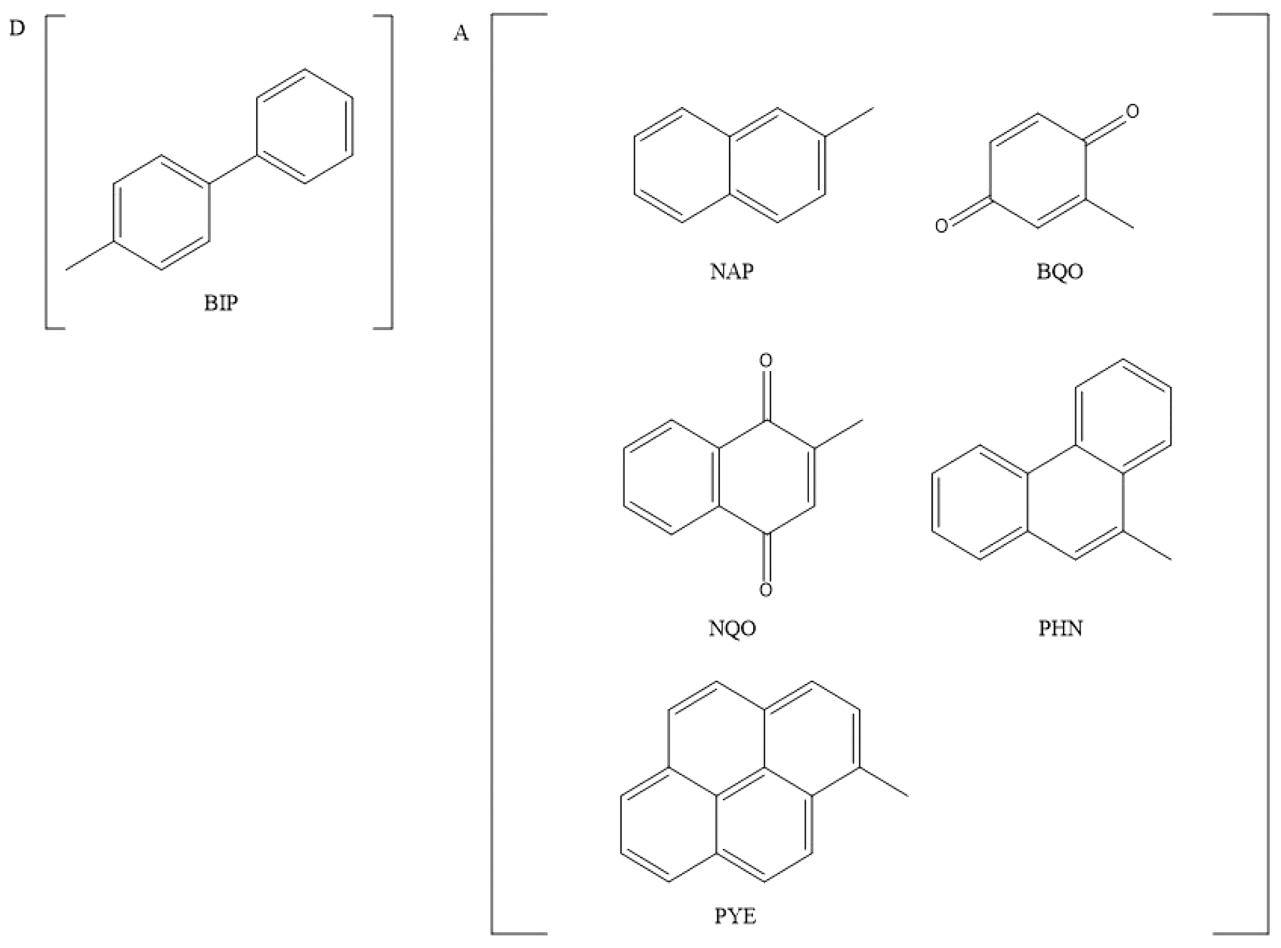
Donor and acceptors group: BIP = 1,1-biphenyl, BQO = 2-benzoquinonyl, NAP = 2-naphthyl, NQO = 2-naphtoquinonyl, PHN = 9-phenanthryl, PYE = 1-pyrenyl.

**Table 1:** Standard Free Energy Changes for Each BIP-br-A Pair in Iso-Octane, THF, and DBE, All Energies in eV

	iso-octane	THF			DBE		
A	Δ*G*_fi_^0^ = Δ*G*_eff_^0^	Δ*G*_fi_^0^	Δ*G*_eff_^0^	Δ*G*^#^	Δ*G*_fi_^0^	Δ*G*_eff_^0^	Δ*G*^#^
BQO	–2.4	–2.4	–1.6		–2.4	–1.8	
NAP	0.0	–0.03	+0.7	+0.2	0.0	+0.6	+0.2
NQO	–2.1	–2.1	–1.3		–2.1	–1.6	
PHN	–0.2	–0.1	+0.6	+0.2	–0.1	+0.5	+0.2
PYE	–0.6	–0.5	+0.3	0.04	–0.6	+0.03	0.01

All ET reactions are exothermic when both solvent (*Q*) and molecular (*q⃗*) nuclear coordinates are in their equilibrium conditions, but in the present kinetic model the elementary ET step occurs at fixed *Q*, either at *Q*_0i_, the equilibrium solvent coordinate of the initial state, at
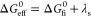
9when Δ*G*_eff_^0^ < 0, or at *Q*_c_, the point at which the potential energy surfaces of the initial and final states cross each other when intramolecular coordinates are kept fixed at their initial equilibrium value. Thus, to apply the proposed kinetic model, it is first necessary to ascertain whether or not ET requires solvent activation, which in turns requires the evaluation of λ_s_. The latter quantity can be estimated by using several approaches: the Born-Onsager model,^1,41^ molecular dynamics simulations,^42,43^ the nonequilibrium polarizable continuum model,^44^ and microscopic theory of solvent response.^45,46^ Herein, taking advantage of the availability of experimental ET rates at different Δ*G*, λ_s_’s are directly estimated from the experimental rate constants of the extremely exothermic BIP-BQO or BIP-NQO pairs and, from the electronic coupling elements reported in ref (43), upon assumption, which can be easily verified a posteriori, that those ET processes do not require solvent activation, i.e. Δ*G*_eff_^0^ < 0. The adopted approach has the limitation that λ_s_ does not depend on the specific acceptor, but it yields reasonable results for all of the A/D pairs considered here, see infra, because, as noted previously by Parson,^43^ in those systems, λ_s_ is weakly dependent on the acceptor.

The above procedure, see the caption of Figure 2 for more details, yields λ_s_ = 0.75 eV and λ_s_ = 0.53 eV for THF and DBE, respectively, obtained as averages between the λ_s_ of the two exothermic BIP-BQO and BIP-NQO pairs (0.86 and 0.63 eV in THF and 0.58 and 0.47 eV in DBE, for BIP-BQO and BIP-NQO, respectively). Estimated solvent reorganization energies are in reasonable agreement with those obtained by Parson using quantum mechanics/molecular dynamics (QMMD) simulations^43^ and compare very well with those estimated by Closs and co-workers, fitting experimental data in THF with a Marcus like rate expression with a single high frequency quantum mode.^21^ Estimated λ_s_’s are plotted against solvent dipole moments in Figure S2; a remarkable linear dependence has been found.

**Figure 2:**
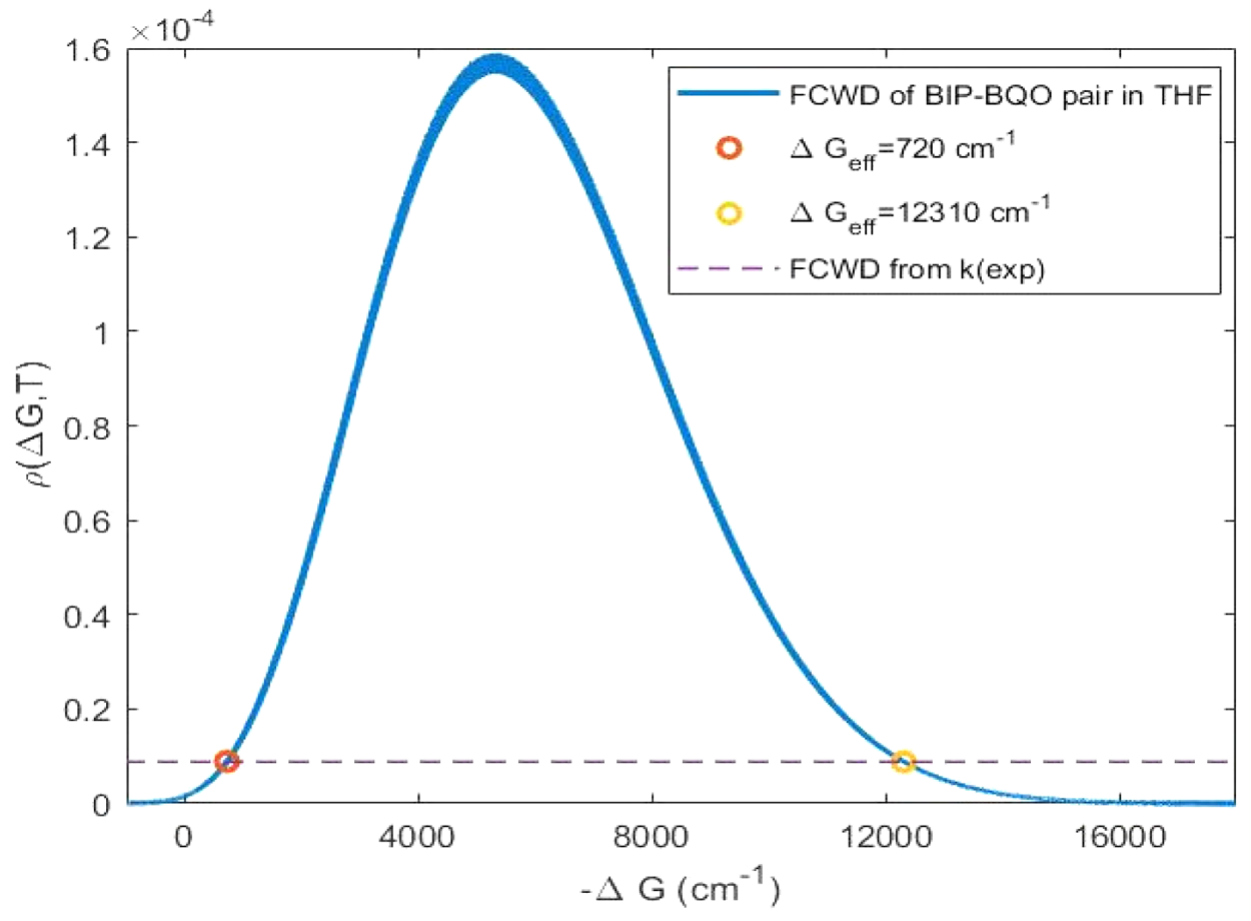
Evaluation of λ_s_ from experimental rates: ρ(Δ*G*, *T*) of BIP-BQO pair in THF (blue curve) is plotted against Δ*G*_fi_^0^. The dashed horizontal line indicates the value of ρ for which, using the electronic coupling element reported in ref (43), eqs 5 and 7 yield the observed rate constant. Two different values of Δ*G*_fi_^0^ (red and yellow circles) are possible, but only that corresponding to the yellow circle is physically sound and leads to Δ*G*_eff_^0^ = −1.53 eV and λ_s_= 0.86 eV. The other value (red circle) is disregarded because it would lead to vanishingly small ET rates for all of the other acceptors (λ_s_ ≈ 2.1 eV).

For the nonpolar iso-octane solvent, the above procedure yields λ_s_’s vanishingly small for all redox pairs, as it occurs in photosynthetic reaction centers,^47^ so that in iso-octane, Δ*G*_eff_^0^ = Δ*G*_fi_^0^. All of the calculated rate constants in iso-octane are reported in Table 2, together with experimental ones. For NQO, the calculated ET rate significantly disagrees with the experimental value, but, as already noted in previous papers,^21,43,48^ this strongly exergonic ET process could also involve an electronically excited state. Indeed, if the ET rate is evaluated at Δ*G*_fi_^0^ corresponding to the first excited states of the NQO anion, obtained by TDDFT computations, keeping fixed the electronic coupling element, a reasonable agreement with the experimental value is found, see Table 2. According to TDDFT results, also ET from BIP to BQO could involve the first electronically excited state of BQO, but in this case better agreement with the experimental rate is obtained for ET into the ground state, see Table 2.

**Table 2:** Experimental and Calculated ET Rate Constants for Each BIP-br-A Pair in Iso-Octane, THF, and DBE, All Constants in s^–1^

	iso-octane		THF		DBE	
A	exp	theo	exp	theo	exp	theo
BQO	3.6 ± 0.8e+06	5.4e+06	2.5 ± 0.3e+08	1.4e+08	3e+07	1.9e+07
		1.0e+08^a^				
NAP	1.5 ± 0.5e+09	2.6e+09	1.5 ± 0.5e+06	2.6e+06	2e+07	3.4e+07
NQO	>2e+09	2.9e+06	3.8 ± 1e+08	8.3e+08	2e+08	2.9e+08
		4.4e+09^a^				
PHN	>2e+09	1.9e+09	1.25 ± 0.2e+07	1.3e+07	4e+08	5.5e+07
PYE	>2e+09	1.3e+10	1.5 ± 0.5e+09	2.9e+09		6.4e+09

a Corresponding to ET into the first excited state of A.

For most of the A/D systems in THF and DBE, Δ*G*_eff_^0^ is positive, see Table 1, so that ET requires an activation step. ET rates are therefore determined using Pauli’s master equation approach, solving the system of differential equations associated with the kinetic scheme of Scheme 1, with *k*_act_ and *k*_ET_ calculated from eqs 1–5, and *k*_dact_ taken from experimental time dependent spectroscopic measurements: *k*_dact_ = 7 × 10^11^ and *k*_dact_ = 8 × 10^10^, for THF and DBE, respectively.^23,49^ The *k*_0_ of eq 1 has been taken as a solvent dependent adjustable parameter; satisfying agreement with experimental results has been obtained by setting *k*_0_ = 5 × 10^12^ and *k*_0_ = 4 × 10^11^ for THF and DBE, respectively.

Calculated ET rate constants in THF and DBE are reported in Table 2 together with experimental ones, and in Figures 3 and 4, where theoretical and experimental rate constants are reported as a function of Δ*G* of ET reactions. According to experimental observations, there is an initial rise of the rates as Δ*G*^0^ decreases, followed by an inverted region, as predicted by Marcus’ theory, where a further decrease of the Δ*G*^0^ leads to a decrease in rate.

**Figure 3:**
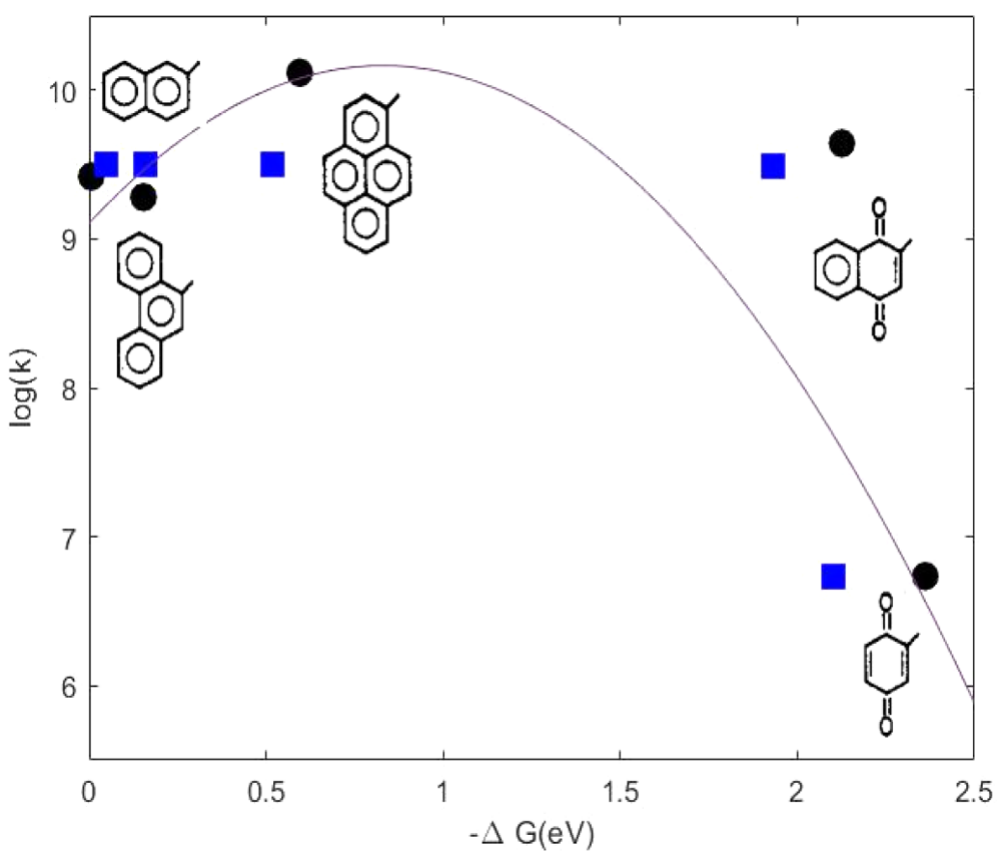
Calculated (black circles) and experimental (blue squares) ET rate constants in iso-octane for each DA pair.

**Figure 4:**
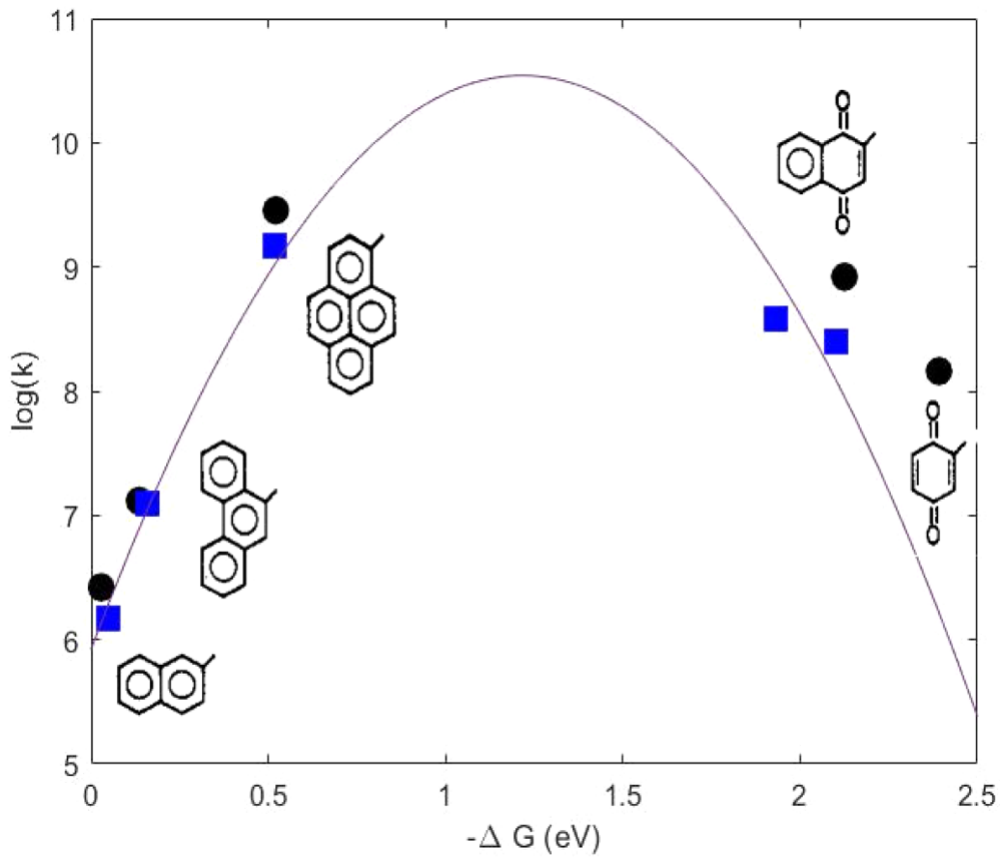
Calculated (black circles) and experimental (blue squares) ET rate constants in THF for each DA pair.

Finally, we come to the most delicate point of the temperature dependence of ET, for which experimental results are available only for two acceptors, BQO and NAP in THF.^22,48^ For the BIP/BQO pair, Δ*G*_eff_^0^ < 0, and ET does not require a solvent activation step, whereas ET in the BIP/NAP pair needs activation by solvent motion. The calculated rates at different *T* are reported in Figure 5, which clearly highlights the differences between the two ET processes: the temperature dependence of ET from BIP to NAP is dominated by the Arrenhius exponential factor, which makes ET rates change by over 4 orders of magnitude by changing *T* from 180 to 400 K. ET from BIP to BQO occurs by tunneling, and since the temperature dependence of ρ(Δ*E*, *T*) is modest, ET is almost independent of temperature. Although our treatment has neglected important factors, such as the temperature dependence of Δ*G*_eff_^0^ and λ_s_,^50^ which proved to be crucial for simulating the unusual temperature dependence of ET rates observed in the fullerene-porphyrin dyad,^4^ the proposed approach provides a satisfying picture of the *T* dependence of ET. It is noteworthy that the dielectric constant of THF exhibits a significant temperature dependence, which, in the range −78 to 30°, can be expressed by the following relation: ϵ(*T*) = −1.50 + 2650/*T*.^51^ Since ϵ(*T*) increases as *T* decreases, λ_s_ will also increase, increasing the ET rates, see Figure 2. Thus, a more refined treatment, such as those outlined in refs (45 and 46), could further improve the agreement with experimental data.

**Figure 5:**
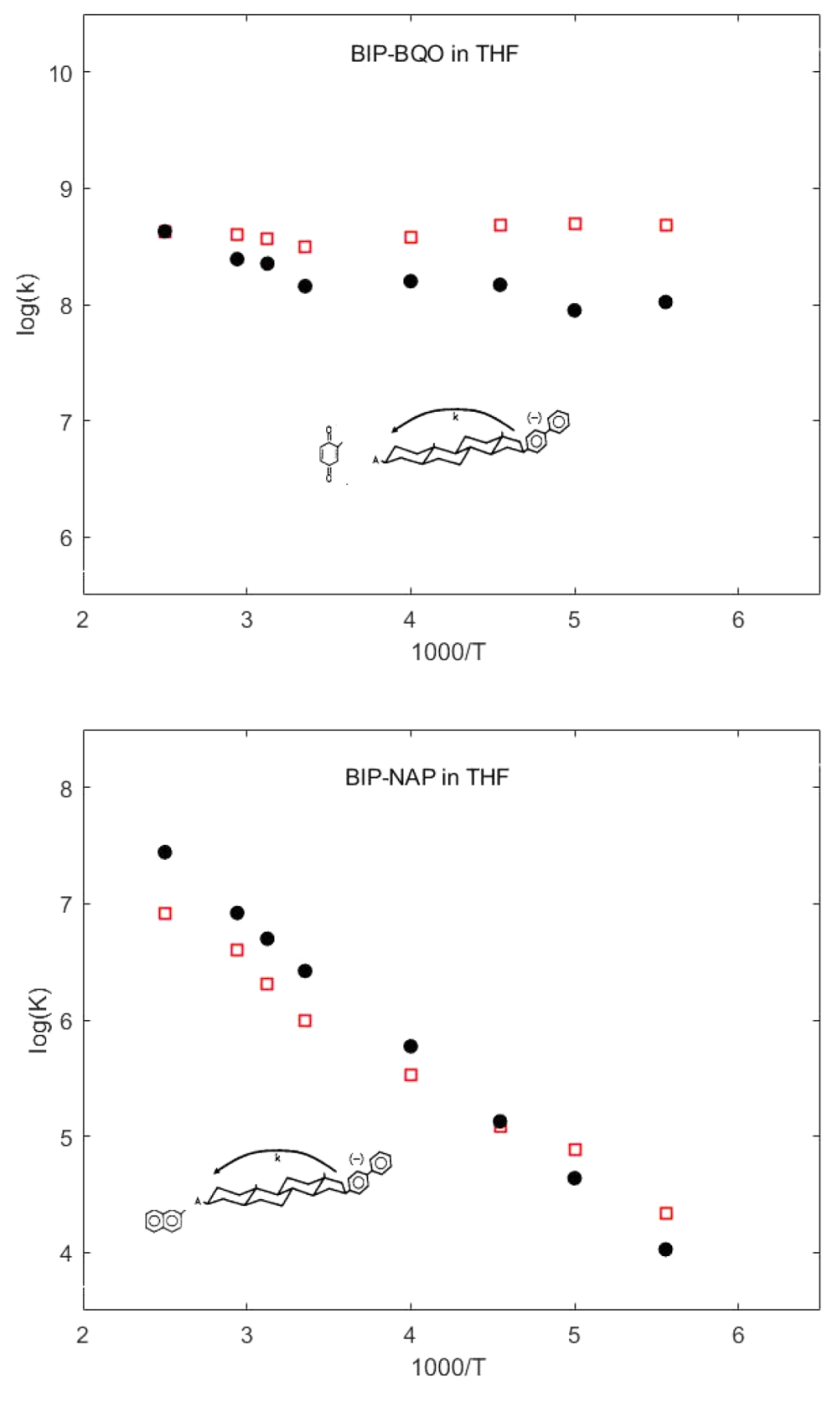
Calculated (black circles) and experimental (red empty squares) temperature dependence of the ET rate constant from BIP^–^ to BQO (top) and NAP (bottom) in THF. The dependence of the dielectric constant on temperature, see text, has been neglected.

We have presented a simple generalization of Marcus’ theory, which allows the inclusion of the whole heat bath provided by intramolecular coordinates of the redox pair in the evaluation of the tunnelling contribution to ET rates, a crucial task for properly handling vibronic degeneracy. In the proposed approach, the elementary ET step occurs always by tunneling, which can be or not be triggered by the environment, according to energy conditions: for highly exothermic ET processes, for which the Δ*G*^0^ of ET overcomes the reorganization energy of the environment, the initial and final states are in vibronic resonance, and ET can occur by tunneling at frozen environmental coordinates. Vice versa, when the energy stabilization of a polar environment is larger than the ET free energy variation, environmental motion is required to trigger ET tunneling, and the whole process is described by a multistep mechanism, in which also the rates of solvent stabilization of a nonequilibrium charge distribution play an important role. The faithful reproduction of the ET temperature dependence in two different cases testifies to the versatitility of the proposed mechanism; of course, further applications are needed for a better assessment of its general applicability.

Most of this work has been stimulated by a series of recent papers by Parson, who used QMMD simulations to compute ET rates.^43,52−54^ Our approach avoids the use of QMMD simulations, relying on the separated treatment of the nuclear motion of the environment from that of the redox pair, whose interplay is treated, when necessary, at the Pauli master equation level. That is not a great limitation; cases in which coherent effects play a role have been successfully addressed using Pauli master equations in the past.^18^ In principle, the approach could represent a physically sound alternative to QMMD simulations, and indeed a significant improvement has been found, if enough experimental data are available, since two quantities, the solvent reorganization energies and *k*_0_, have to be evaluated from experimental data; in practice, when experimental data are scarce, it is still necessary to find a valid approach to evaluate from first-principles λ_s_ and *k*_0_. Work is in progress along this line. The approach also provides a significant improvement with respect to nonadiabatic theories developed in the past, for the most exothermic ET reactions in a nonpolar solvent.^21^

Although the procedure is entirely numerical, the approach offers a mechanistically very clear picture of the ET process. The proposed multistep mechanism also shares common facets with other recently published mechanistic pictures: the flickering resonance^55^ and the unfurling mechanisms^56^ are both based on the assumption that transient degeneracy among different redox species triggers coherent charge motion.

## Computational Methods

Franck–Condon weighted densities of states have been computed by using a development version of the MolFC package, available on request. The internal (curvilinear) representation of normal coordinates has been adopted in all of the cases.^57^ Equilibrium geometries, normal coordinates, and vibrational frequencies in the neutral and anionic forms were computed at the density functional theory (DFT) level by using the B3LYP functional with the 6-31+G(d,p) basis set. Excited state energies have been obtained by time dependent DFT computations, using the same functional and basis set. In all of the computations, the solvent effect was included by using the equilibrium polarizable continuum model (PCM). The electronic coupling elements reported in ref (43) have been employed throughout. The set of coupled ODEs has been solved by using the Dormand–Prince method of order 4, a member of the Runge–Kutta family of ODE solvers, as implemented in the MATLAB package.^58^

## Supporting Information Available

Computed λ_i_’s, normal-mode frequencies, and equilibrium position displacements; derivation of eq 2; plot of λ_s_ vs solvent dipole moment; evaluation of λ_s_ from experimental rates; thermally averaged Franck–Condon weighted density of states in different solvents (ZIP)
