# Supplementary material for: Electron Transfer Rates in Polar and Non-Polar Environments: a Generalization of Marcus’ Theory to Include an Effective Treatment of Tunneling Effects
Source: J Phys Chem Lett. 2022 Sep 27;13(39):9148–55. doi: 10.1021/acs.jpclett.2c02343 (PMC9549518; doi:10.1021/acs.jpclett.2c02343)
Supplement: Supplementary file 1 — jz2c02343_si_001.zip [file jz2c02343_si_001.zip › SI_ET_rates/SI_ET_rates_def.pdf]

Electron Transfer Rates in Polar and Non-Polar Environments: a Generalization of Marcus' Theory to Include an Effective Treatment of Tunneling Effects

Anna Leo and Andrea Peluso*

Dipartimento di Chimica e Biologia

Università di Salerno, I-84084 Fisciano, Salerno, Italy

E-mail: apeluso@unisa.it

Supporting Information

*

Table S1: Computed λ_i of all molecules from gas phase DFT calculations (gas) and by using harmonic approximation (h.a.) and the displacement vectors reported in Table S2. All energies are in eV.

Molecule	λ_i gas	λ_i h.a. THF	λ_i h.a. DBE	λ_i h.a. ISO
BIP	0.31	0.29	0.29	0.29
BQO	0.27	0.26	0.26	0.26
NAP	0.12	0.12	0.11	0.11
NQO	0.24	0.23	0.23	0.23
PHN	0.15	0.15	0.15	0.15
PYE	0.11	0.10	0.10	0.10

Table S2: Wavenumber ($\tilde{\omega}$ in cm^{-1}) and equilibrium position displacement **K** (dimesionless units) of the most displaced normal modes upon ET.

Molecule	Iso-octane		THF		DBE	
	$\tilde{\omega}$	K	$\tilde{\omega}$	K	$\tilde{\omega}$	K
BIP	66.20	5.638	67.26	5.637	66.93	5.592
	312.6	-0.622	312.9	-0.586	312.7	-0.610
	420.9	0.543	421.3	0.528	421.1	0.534
	1014	-0.596	1013	-0.605	1014	-0.595
	1304	0.619	1303	0.633	1303	0.627
	1648	-0.796	1645	-0.801	1647	-0.799
BQO	467.7	1.712	470.1	1.653	468.9	1.685
	821.6	0.650	824.6	0.652	822.9	0.652
	1152	-0.561	1161	-0.537	1157	-0.549
	1484	-0.661	1477	-0.709	1481	-0.684
	1649	0.933	1652	-0.929	1650	-0.931
NAP	510.9	-0.940	508.0	-0.944	509.5	-0.939
	1377	-0.699	1373	-0.699	1375	-0.700
	1594	0.553	1593	0.573	1593	0.561
NQO	365.8	-0.647	364.9	-0.586	365.9	-0.627
	462.1	1.336	463.4	1.301	463.0	1.318
	1632	0.897	1631	0.898	1631	0.898
PHN	95.71	0.713	-	-	80.26	-0.607
	406.1	-0.891	405.2	-0.905	405.7	0.894
	1630	0.732	1627	0.726	1629	-0.731
PYE	407.5	-0.803	407.3	-0.821	407.4	-0.810

Derivation of Equation 2

Let us denote by Q the scalar variable proportional to a certain component of the polarization vector and by $\mathbf{q}$ the vector of intramolecular coordinates. We follow Marcus' assumptions that the potential energy surfaces of the initial and final states are quadratic diagonal functions in Q and $\mathbf{q}$:

$$V_i(\mathbf{q}, \mathrm{Q}) = \frac{1}{2}\mathbf{q}^\dagger \mathrm{a}\mathbf{q} + \frac{1}{2}\mathrm{K}\mathrm{Q}^2 \tag{1}$$

$$V_f(\mathbf{q}, \mathrm{Q}) = \frac{1}{2}(\mathbf{q} - \mathbf{q}_{0f})^\dagger \mathrm{b}(\mathbf{q} - \mathbf{q}_{0f}) + \frac{1}{2}\mathrm{K}(\mathrm{Q} - \mathrm{Q}_{0f})^2 + \Delta G^0_{fi} \tag{2}$$

where $\mathbf{q}_{0f}$ and Q_{0f} denote the position of the minima in the final state, the quantities $\frac{1}{2}\mathbf{q}^\dagger_{0f}\mathrm{b}\mathbf{q}_{0f}$ and $\frac{1}{2}\mathrm{K}\mathrm{Q}^2_{0f}$ represent λ_i and λ_s, respectively, and ΔG^0_{fi} is the free energy change of ET reaction.[1] From Eq. 2 it is easily seen that if ET occurs by tunnelling at Q=0 the effective free energy change is:

$$\Delta G^0_{eff} = \Delta G^0_{fi} + \lambda_s. \tag{3}$$

For solvent activated ET, the activation energy is found by imposing the condition $V_i(Q_c, \mathbf{q})$ = $V_f(Q_c, \mathbf{q})$ at $\mathbf{q} = 0$. That is:

$$\frac{1}{2}\mathrm{K}\mathrm{Q}_c^2 = \lambda_i + \frac{1}{2}\mathrm{K}(\mathrm{Q}_c - \mathrm{Q}_{0f})^2 + \Delta G^0_{fi} \tag{4}$$

which yields:

$$\mathrm{Q}_c = \frac{\Delta G^0_{eff} + \lambda_i}{\mathrm{K}\mathrm{Q}_0}, \tag{5}$$

and

$$G^{\#} = \frac{1}{2}\mathrm{K}\mathrm{Q}_c^2 = \frac{(\Delta G^0_{eff} + \lambda_i)^2}{4\lambda_s}$$

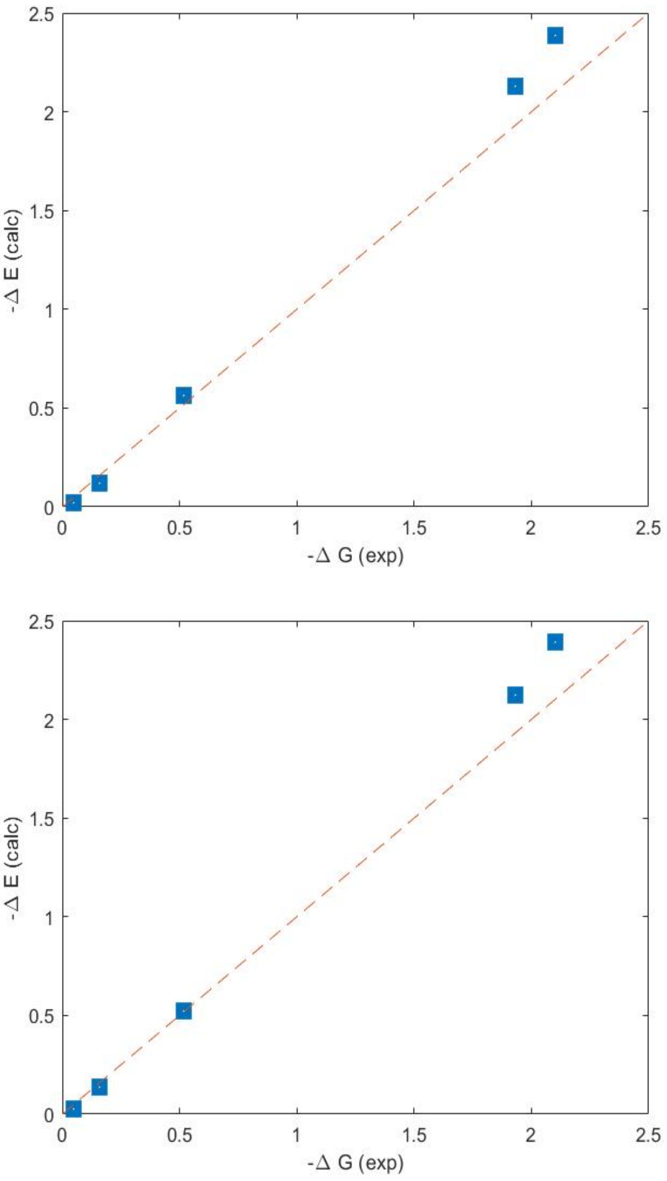

Figure S1: Calculated energy differences for ET from BIP^- to NAP, PHN, PYE, NQO and BQO (in order of increasingly negative ΔE) against experimental ΔG values in DBE (top panel) and THF (bottom panel). All energies in eV

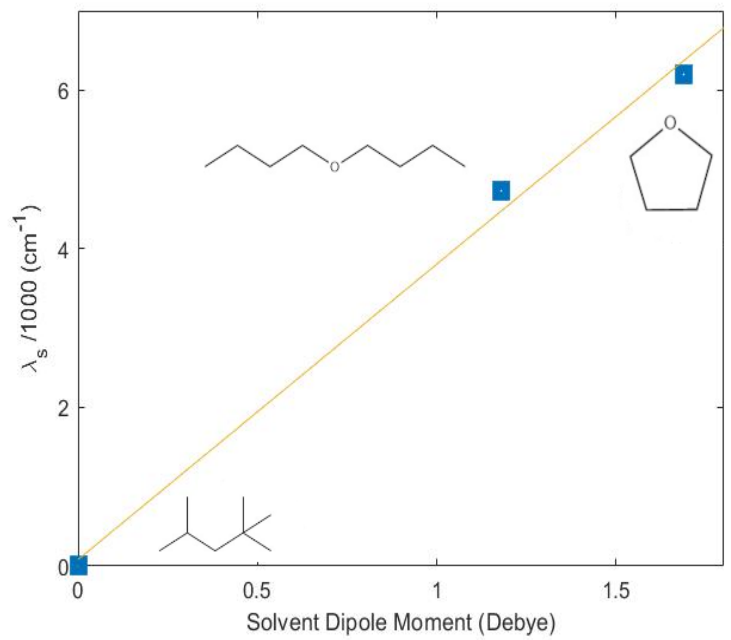

Figure S2: Estimated solvent reorganization energies plotted against dipole moment of iso-octane, DBE and THF.

References

(1) Sumi, H.; Marcus, R. A. Dynamical Effects in Electron Eransfer Reactions. *J. Chem. Phys.* **1986**, *84*, 4894–4914.

(2) Parson, W. W. Effects of Free Energy and Solvent on Rates of Intramolecular Electron Transfer in Organic Radical Anions. *J. Phys. Chem.* **2017**, *121*, 7297–7306.

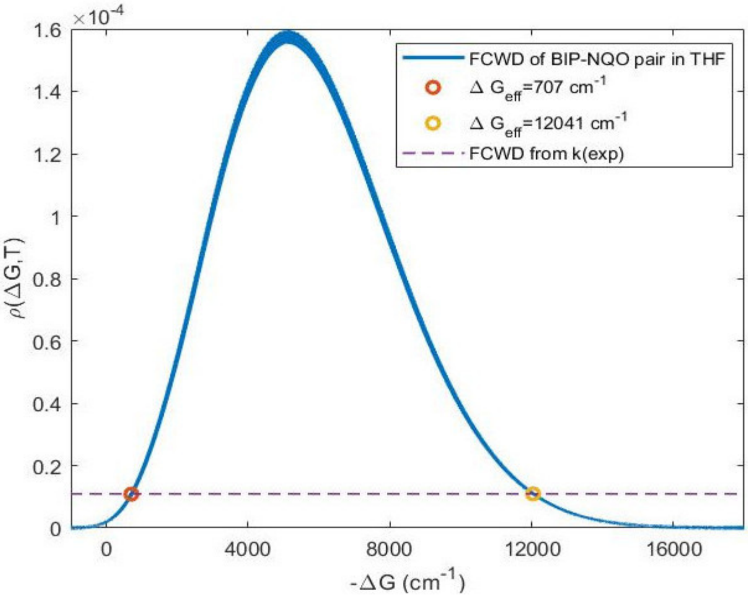

Figure S3: Evaluation of λ_s from experimental rates: $\rho(\Delta G, T)$ of BIP-NQO pair in THF (blue curve) is plotted against ΔG^0_{fi}. The dashed horizontal line indicates the value of ρ for which, using the electronic coupling element reported in ref. 2, $\rho(\Delta G, T) = k_{exp}/(2\pi|V_{f1}|^2/\hbar)$. The equality occurs at two different values of ΔG^0_{fi} (red and yellow circles). Only the ΔG value corresponding to the yellow circle is physically sound and leads to $\Delta G^0_{eff} = -1.49$ eV and λ_s= 0.63 eV. The other value (red circle) is disregarded because it would lead to vanishingly small ET rates for all the other acceptors.

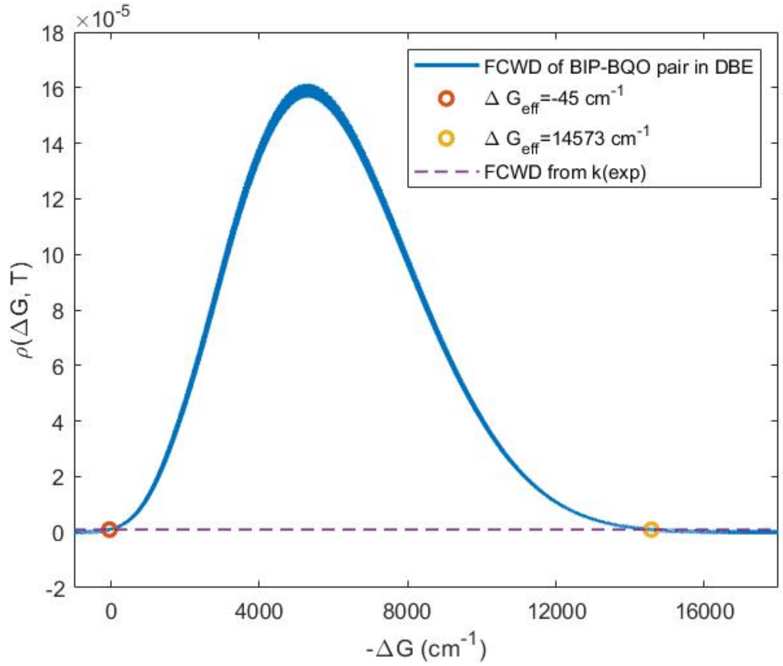

Figure S4: Evaluation of λ_s from experimental rates: $\rho(\Delta G, T)$ of BIP-BQO pair in DBE (blue curve) is plotted against ΔG^0_{fi}. The dashed horizontal line indicates the value of ρ for which, using the electronic coupling element reported in ref. 2, $\rho(\Delta G, T) = k_{exp}/(2\pi|V_{f1}|^2/\hbar)$. The equality occurs at two different values of ΔG^0_{fi} (red and yellow circles). Only the ΔG value corresponding to the yellow circle is physically sound and leads to $\Delta G^0_{eff} = -1.80$ eV and λ_s= 0.58 eV. The other value (red circle) is disregarded because it would lead to vanishingly small ET rates for all the other acceptors.

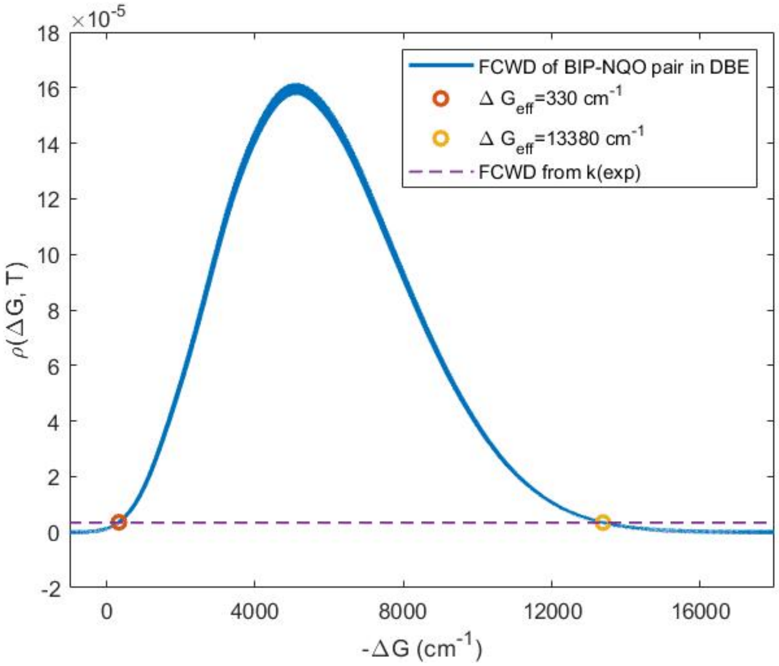

Figure S5: Evaluation of λ_s from experimental rates: $\rho(\Delta G, T)$ of BIP-NQO pair in DBE (blue curve) is plotted against ΔG^0_{fi}. The dashed horizontal line indicates the value of ρ for which, using the electronic coupling element reported in ref. 2, $\rho(\Delta G, T) = k_{exp}/(2\pi|V_{f1}|^2/\hbar)$. The equality occurs at two different values of ΔG^0_{fi} (red and yellow circles). Only the ΔG value corresponding to the yellow circle is physically sound and leads to $\Delta G^0_{eff} = -1.65$ eV and λ_s= 0.47 eV. The other value (red circle) is disregarded because it would lead to vanishingly small ET rates for all the other acceptors.

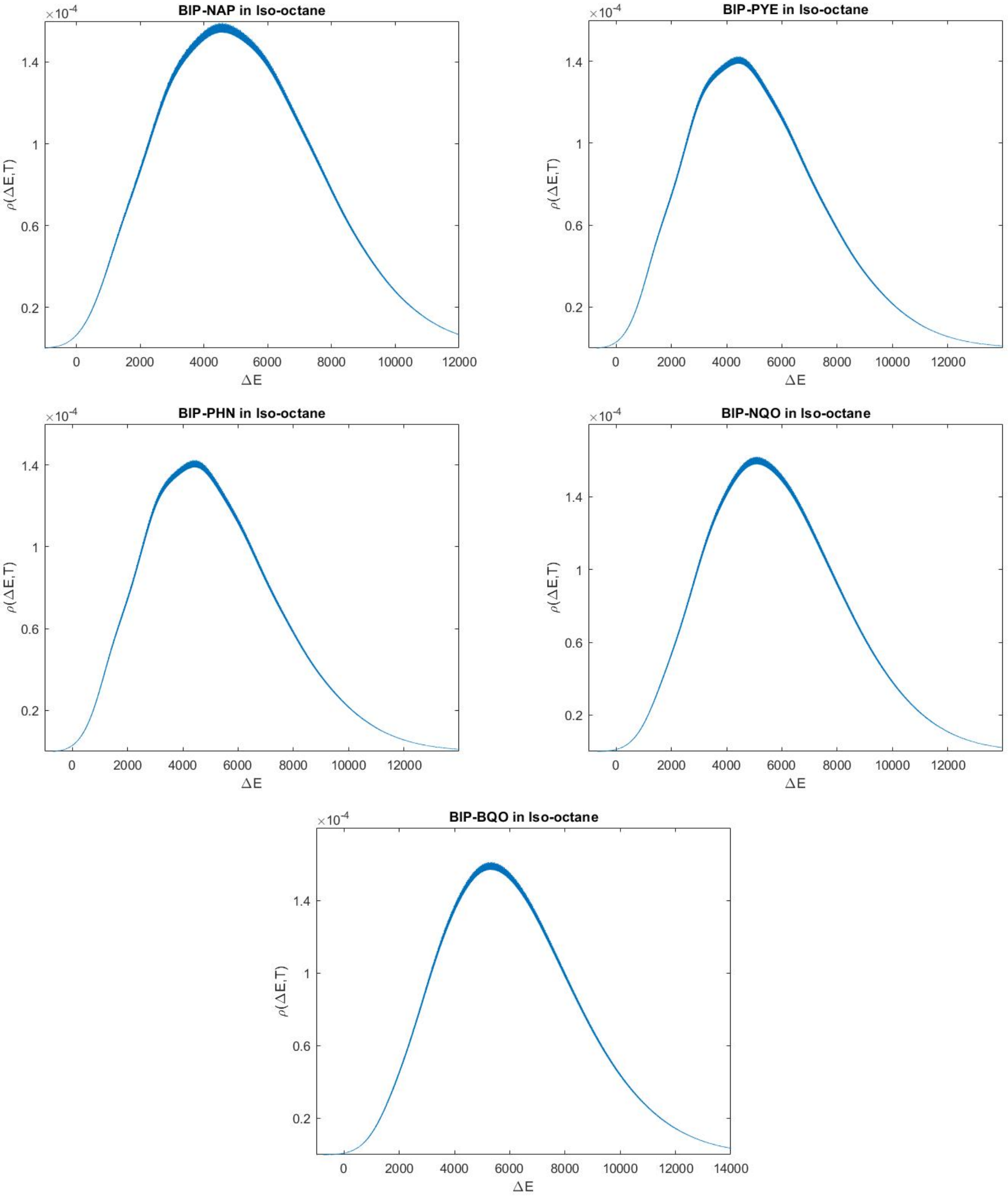

Figure S6: Thermally averaged Franck-Condon weighted density of states (cm) in iso-octane; T=298 K, ΔE cm^{-1}.

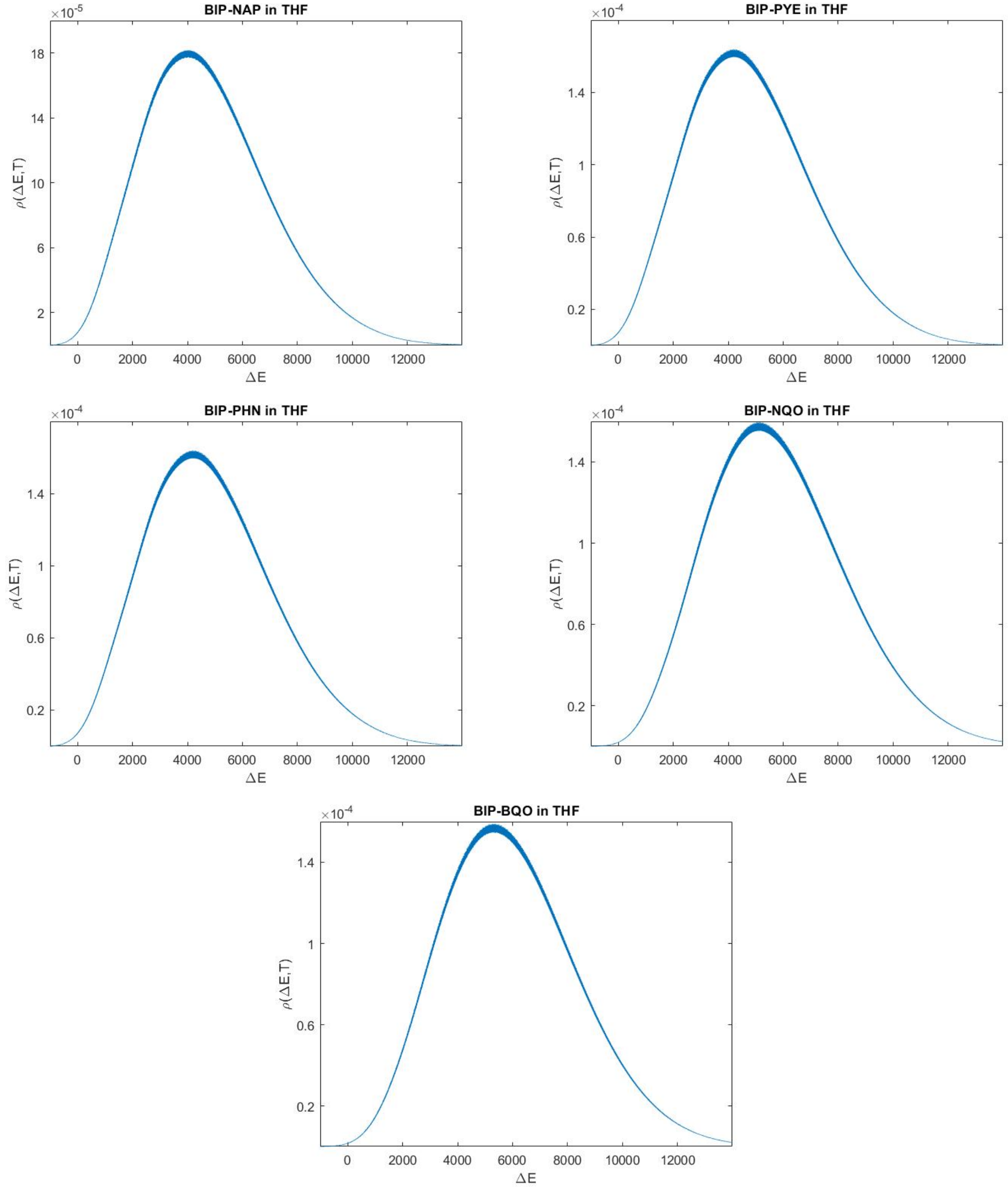

Figure S7: Thermally averaged Franck-Condon weighted density of states (cm) in THF; T=298 K, ΔE cm^{-1}.

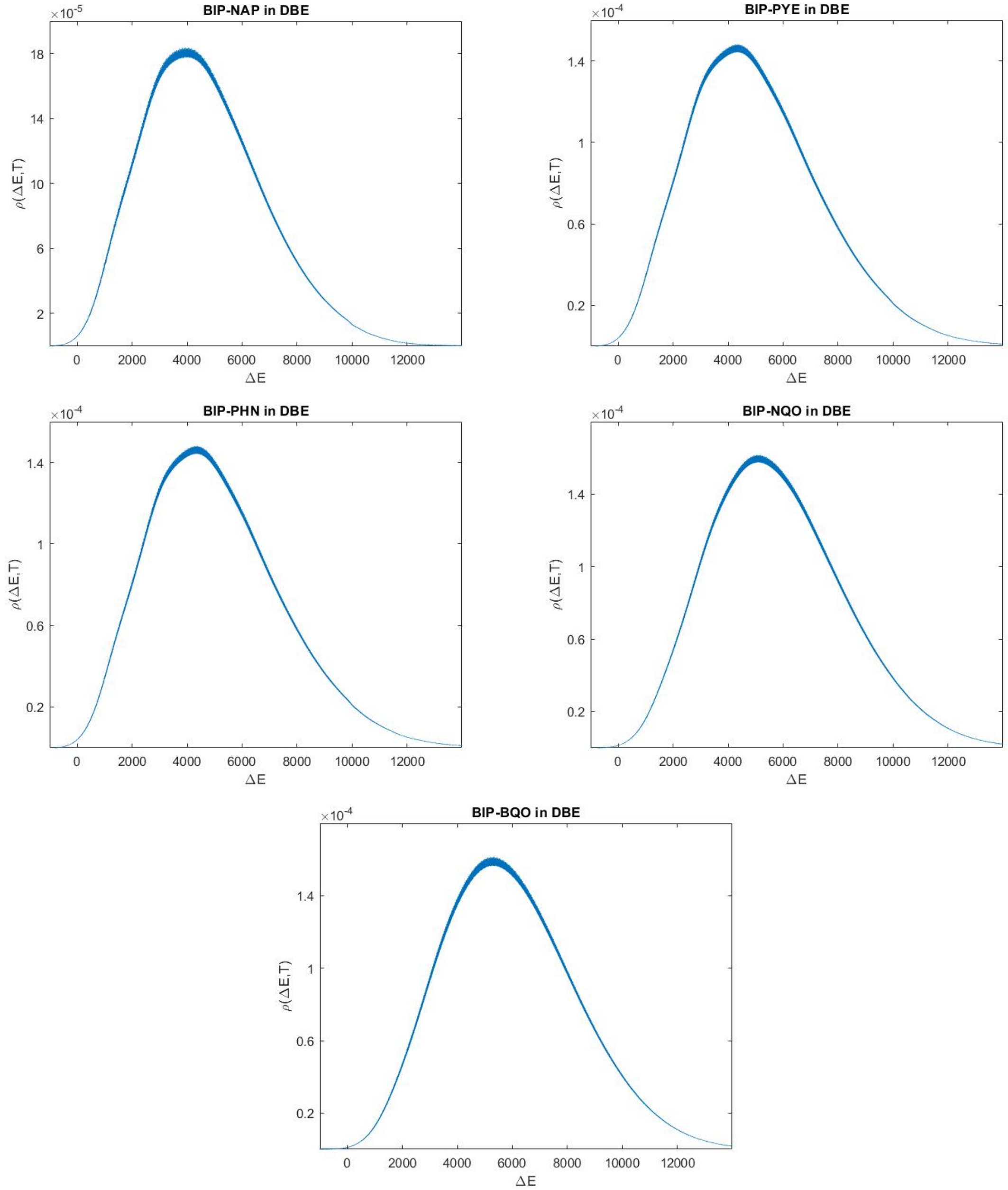

Figure S8: Thermally averaged Franck-Condon weighted density of states (cm) in DBE; T=298 K, ΔE cm^{-1}.
