# Supplementary figures and images for: Electron Transfer Rates in Polar and Non-Polar Environments: a Generalization of Marcus’ Theory to Include an Effective Treatment of Tunneling Effects
Source: J Phys Chem Lett. 2022 Sep 27;13(39):9148–55. doi: 10.1021/acs.jpclett.2c02343 (PMC9549518; doi:10.1021/acs.jpclett.2c02343)

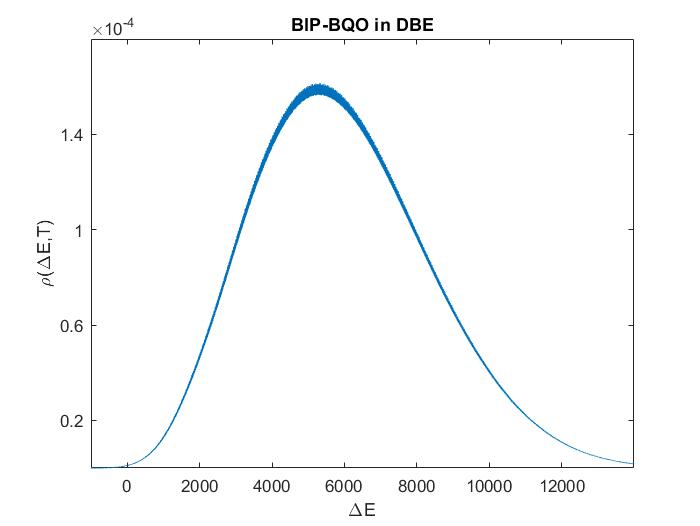

Supplement: Supplementary file 1 — jz2c02343_si_001.zip [file jz2c02343_si_001.zip › SI_ET_rates/conv_bqo_dbe.jpg]

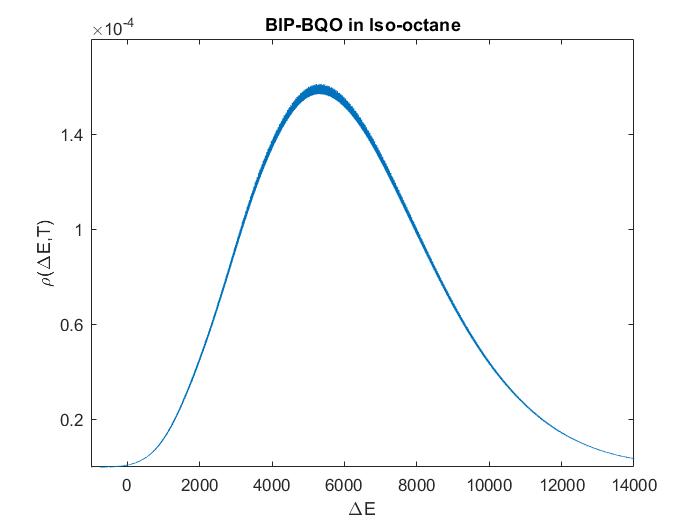

Supplement: Supplementary file 1 — jz2c02343_si_001.zip [file jz2c02343_si_001.zip › SI_ET_rates/conv_bqo_oct.jpg]

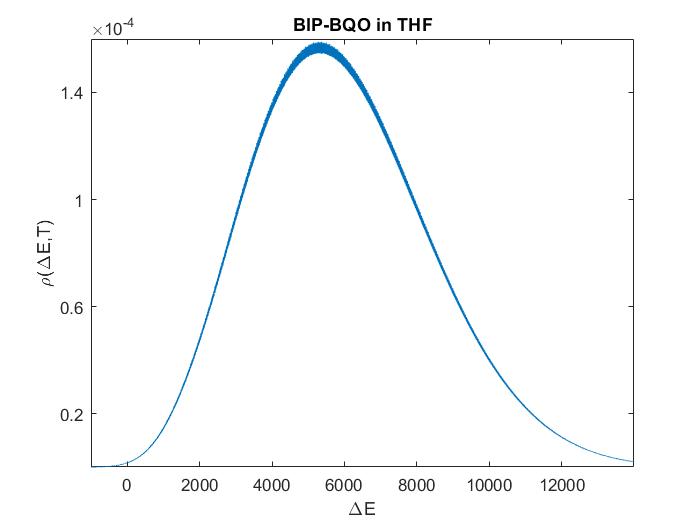

Supplement: Supplementary file 1 — jz2c02343_si_001.zip [file jz2c02343_si_001.zip › SI_ET_rates/conv_bqo_thf.jpg]

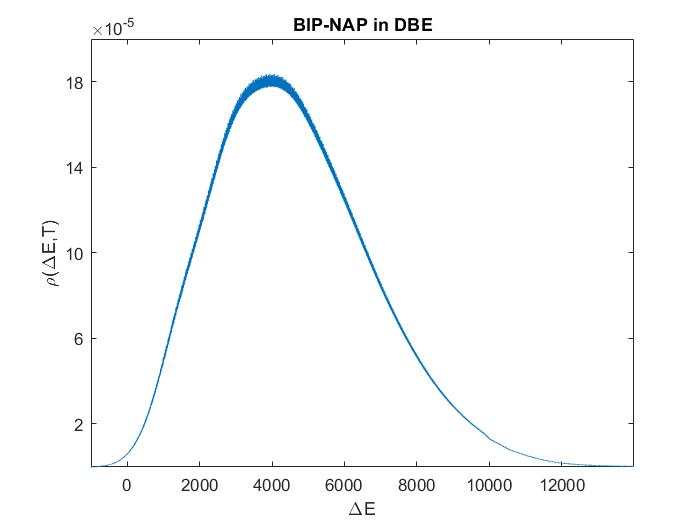

Supplement: Supplementary file 1 — jz2c02343_si_001.zip [file jz2c02343_si_001.zip › SI_ET_rates/conv_nap_dbe.jpg]

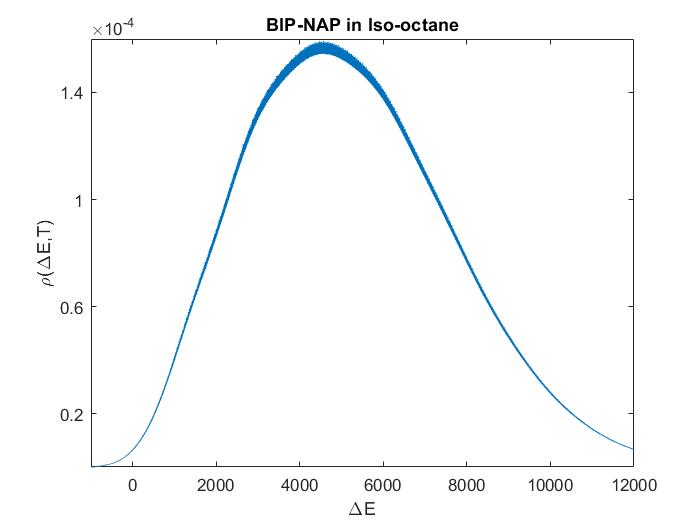

Supplement: Supplementary file 1 — jz2c02343_si_001.zip [file jz2c02343_si_001.zip › SI_ET_rates/conv_nap_oct.jpg]

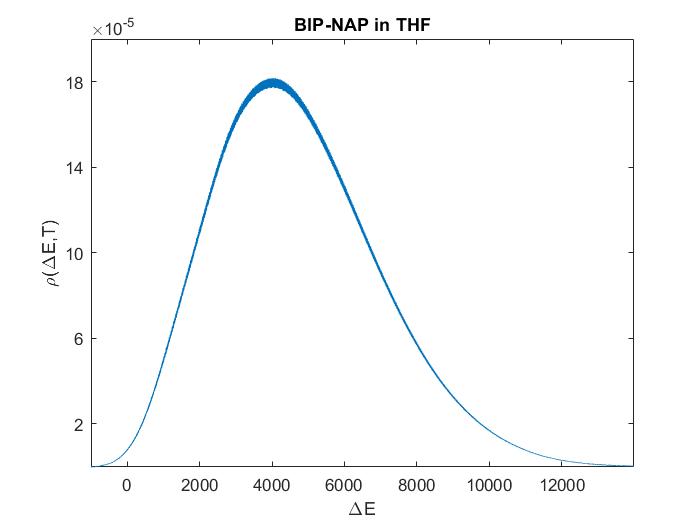

Supplement: Supplementary file 1 — jz2c02343_si_001.zip [file jz2c02343_si_001.zip › SI_ET_rates/conv_nap_thf.jpg]

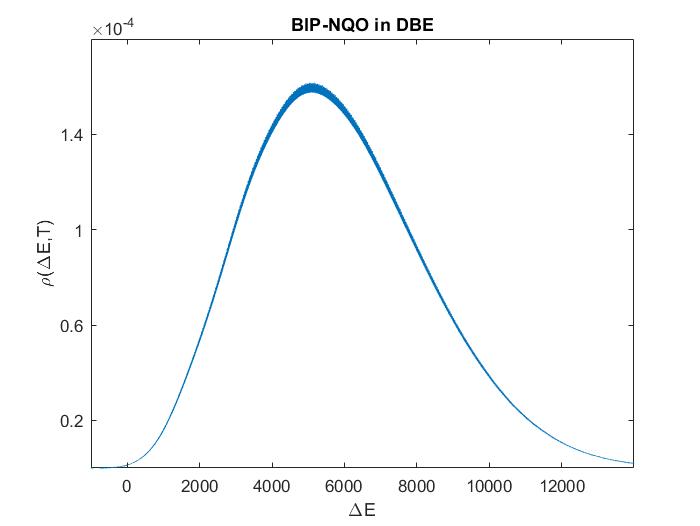

Supplement: Supplementary file 1 — jz2c02343_si_001.zip [file jz2c02343_si_001.zip › SI_ET_rates/conv_nqo_dbe.jpg]

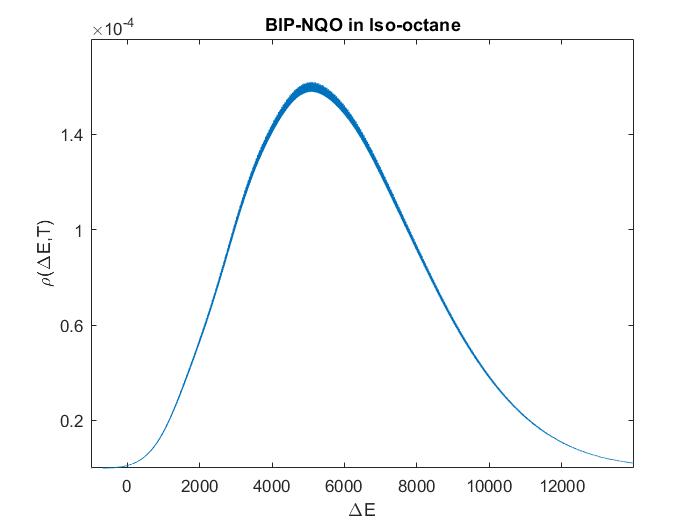

Supplement: Supplementary file 1 — jz2c02343_si_001.zip [file jz2c02343_si_001.zip › SI_ET_rates/conv_nqo_oct.jpg]

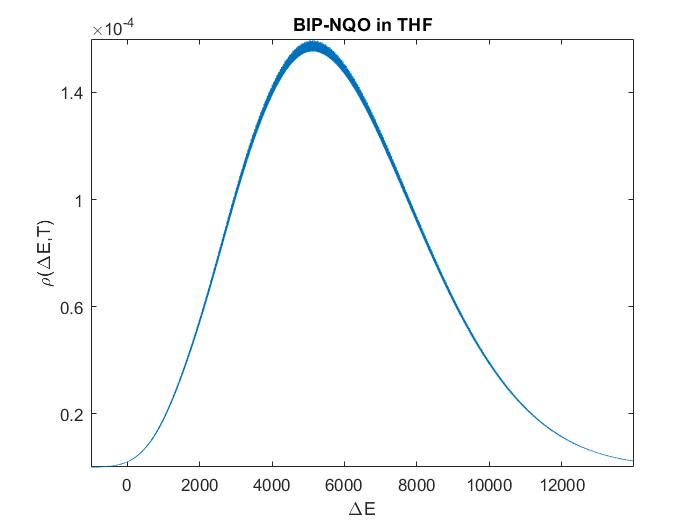

Supplement: Supplementary file 1 — jz2c02343_si_001.zip [file jz2c02343_si_001.zip › SI_ET_rates/conv_nqo_thf.jpg]

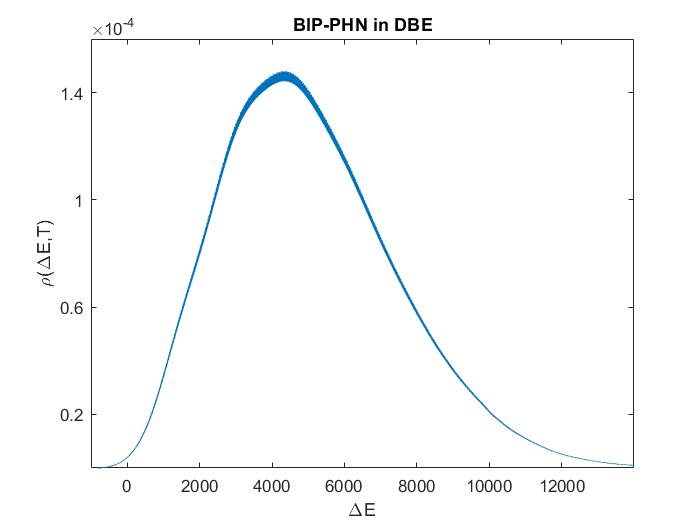

Supplement: Supplementary file 1 — jz2c02343_si_001.zip [file jz2c02343_si_001.zip › SI_ET_rates/conv_phn_dbe.jpg]

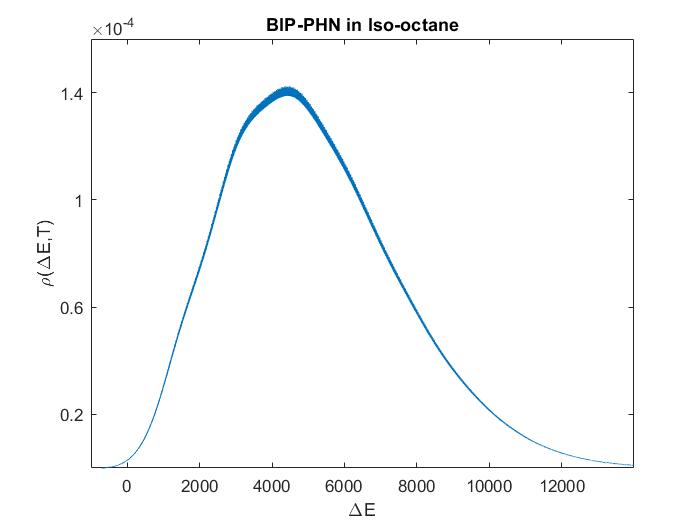

Supplement: Supplementary file 1 — jz2c02343_si_001.zip [file jz2c02343_si_001.zip › SI_ET_rates/conv_phn_oct.jpg]

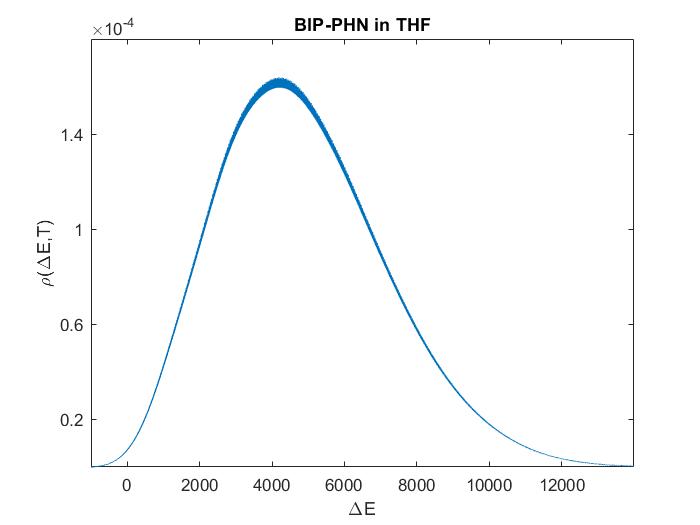

Supplement: Supplementary file 1 — jz2c02343_si_001.zip [file jz2c02343_si_001.zip › SI_ET_rates/conv_phn_thf.jpg]

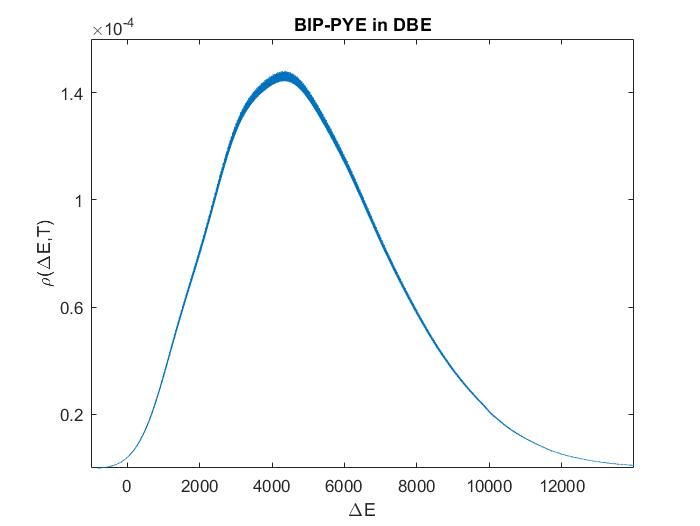

Supplement: Supplementary file 1 — jz2c02343_si_001.zip [file jz2c02343_si_001.zip › SI_ET_rates/conv_pye_dbe.jpg]

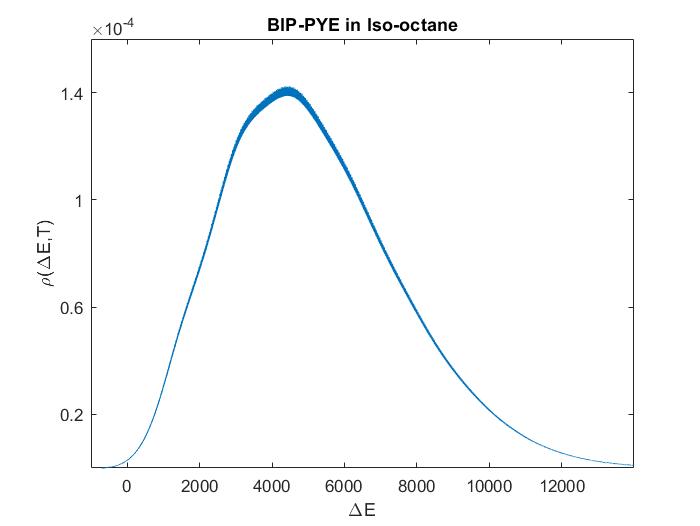

Supplement: Supplementary file 1 — jz2c02343_si_001.zip [file jz2c02343_si_001.zip › SI_ET_rates/conv_pye_oct.jpg]

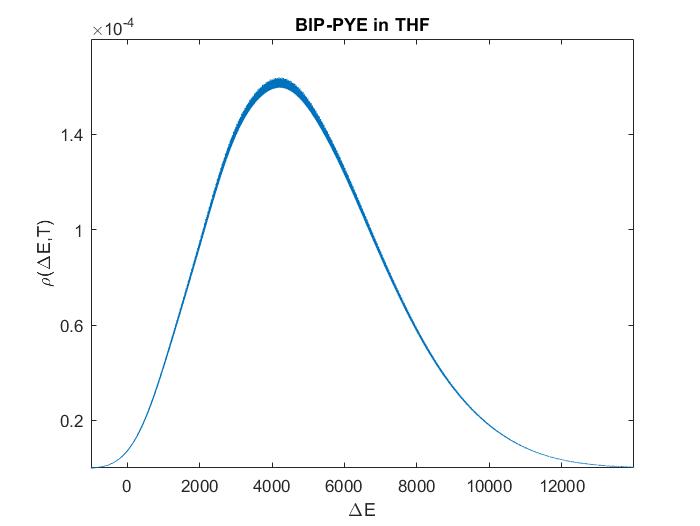

Supplement: Supplementary file 1 — jz2c02343_si_001.zip [file jz2c02343_si_001.zip › SI_ET_rates/conv_pye_thf.jpg]

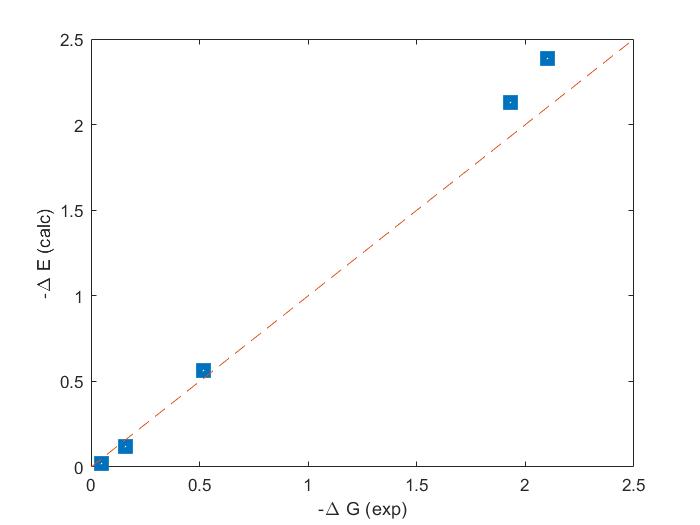

Supplement: Supplementary file 1 — jz2c02343_si_001.zip [file jz2c02343_si_001.zip › SI_ET_rates/deltaG_eth.jpg]

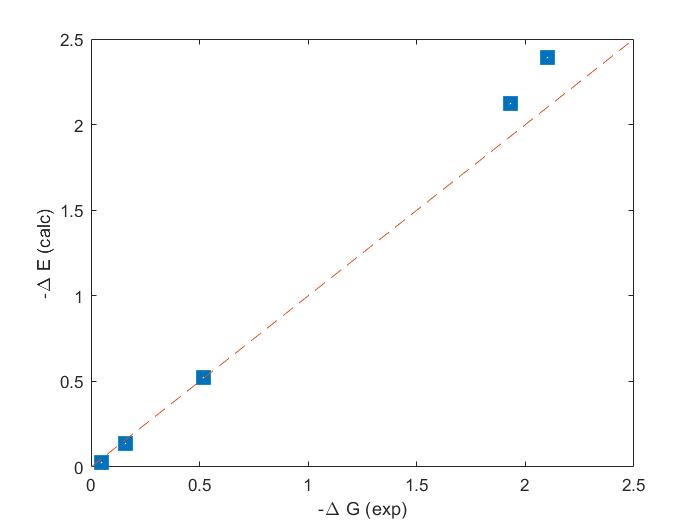

Supplement: Supplementary file 1 — jz2c02343_si_001.zip [file jz2c02343_si_001.zip › SI_ET_rates/deltaG_thf.jpg]

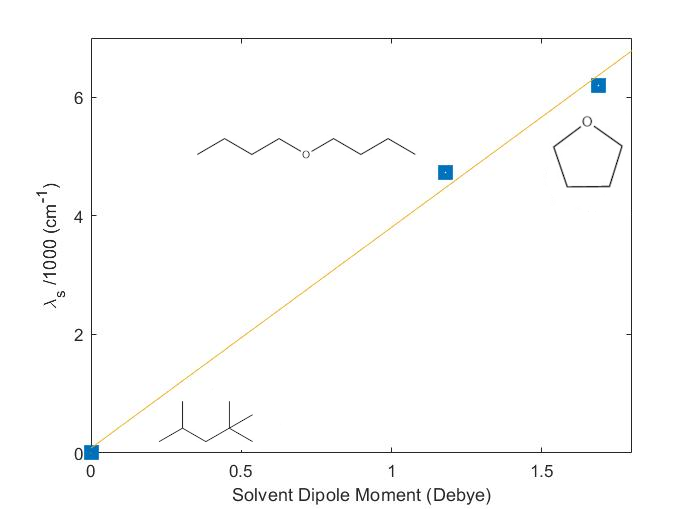

Supplement: Supplementary file 1 — jz2c02343_si_001.zip [file jz2c02343_si_001.zip › SI_ET_rates/lambda_solvents.png]

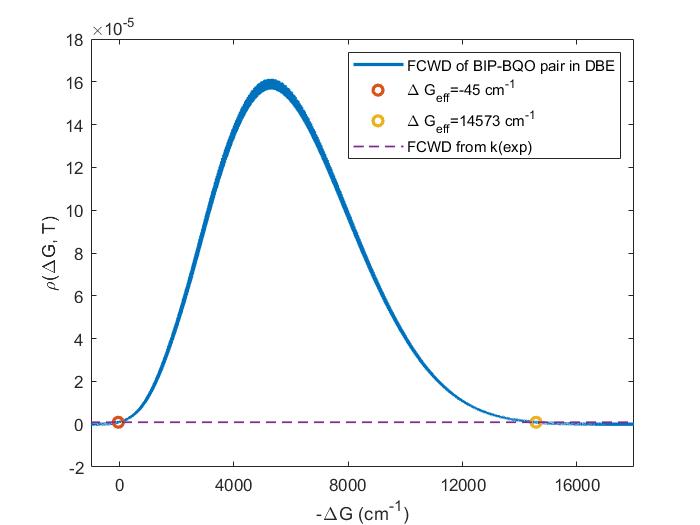

Supplement: Supplementary file 1 — jz2c02343_si_001.zip [file jz2c02343_si_001.zip › SI_ET_rates/new_bqo_fcwd_dbe.jpg]

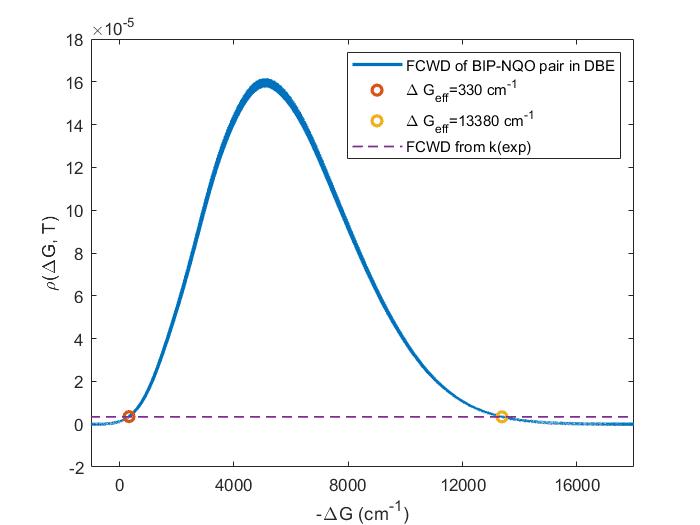

Supplement: Supplementary file 1 — jz2c02343_si_001.zip [file jz2c02343_si_001.zip › SI_ET_rates/new_nqo_fcwd_dbe.jpg]

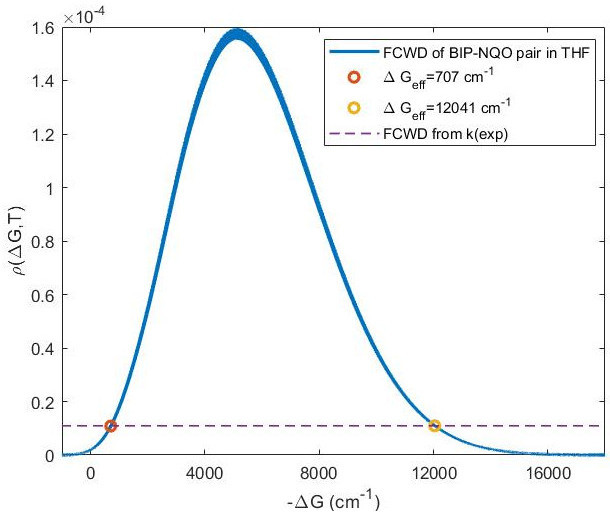

Supplement: Supplementary file 1 — jz2c02343_si_001.zip [file jz2c02343_si_001.zip › SI_ET_rates/new_nqo_fcwd_thf.jpg]
