# Supplementary material for: Electron Transfer Rates in Polar and Non-Polar Environments: a Generalization of Marcus’ Theory to Include an Effective Treatment of Tunneling Effects
Source: J Phys Chem Lett. 2022 Sep 27;13(39):9148–55. doi: 10.1021/acs.jpclett.2c02343 (PMC9549518; doi:10.1021/acs.jpclett.2c02343)
Supplement: Supplementary file 1 — jz2c02343_si_001.zip [file jz2c02343_si_001.zip › SI_ET_rates/Bibfiles/ReviewersComments.docx]

Reviewer: 2

Recommendation: This paper is not recommended because the material is not appropriate for The Journal of Physical Chemistry Letters.

Comments:
This paper allegedly introduces a generalization of Marcus theory to include “tunneling”. A theoretical framework is introduced, and ET transfer rate are calculated and compared to experimental values for D-A systems at solvents of different polarity. 
Unfortunately, I cannot recommend this paper for publication in JPCL, as I see substantial problems in it. These problems include: Inconsistencies in the theoretical formulation, lack of clarity with respect to the calculated entities, and to their fitting to available experimental data, and finally, incorrect statements.

A partial list of specific problems includes:

1.	The statement: “A general and successful quantum mechanics formulation of ET reactions in polarizable media which include tunneling effects is not available yet” seems completely wrong. Even the classical Marcus formula includes the electronic tunneling matrix element. Extensions which account quantum mechanically also for the vibrations are well known, where the simplest ones are based on Fermi’s golden rule (as done here in Eq. 5, but often done in a more consistent and general way than here). This is a textbook level material (e.g. May and Kuhn book on charge and energy transfer).

The assertion that the statement in the manuscript as "completely wrong" suggests that the reviewer does not recognize the serious limitations of the Marcus treatment and does not appreciate the difficulty of fully overcoming these limitations. This is certainly not "textbook level material".

The reviewer's statement that Eq. 5 is based on Fermi's golden rule is obviously incorrect; perhaps he or she meant Eq. 4.

The reviewer does not provide any support for the claim that other well-known treatments of vibrational effects are "more consistent and general." The treatment used in the manuscript is much more general than most previous treatments, and it seems to me to be entirely self-consistent.

There is no attempt to justify the main strategy here, as depicted in scheme 1. Is there any reason to assume that the {D-A}* and {DA-}* species are stable entities on the time scale of 1/k_ET,1/k_act,1/k_deact ? Otherwise, there is no meaning to write the master equations. Any other time-scale separation argument that justifies separating the “activation” from the “ET”? Why is it needed at all?

The manuscript does not assume that {D-A}* and {DA-}* are stable entities. They are transient states that are formed and decay in the course of the reaction. The manuscript addresses how a polarizable medium affects the formation and decay of these states. It is helpful conceptually to treat these steps separately from the quantum mechanical coupling of the two states. I don't see any inconsistencies here.

Interestingly, it is never mentioned if, and if so how, this master equation was used to extract calculated rate constants which are presented as the main output of the theoretical treatment.

I thought the manuscript was clear enough on this point, but perhaps a more detailed explanation would help.

The proposed “activation rates” are calculated by a simple Marcus rate formula, which corresponds to a driving force: Ef-Ei+lamda_i and to reorganization lambda_s. It is not clear at all why that is valid, and even what was the motivation to write this formula. Since {D-A}* is a sub ensemble to {D-A}, they cannot be regarded as orthogonal (complimentary) ensembles, as needed for defining a transition rate between them. It is not even clear what is the alleged process here. It is not a charge transfer event, but what kind of event is it, to merit a Marcus like rate expression ?!

The reviewer again appears not to understand the meaning of {D-A}*. The use of the Marcus expression to find the activation energy for the formation of {D-A}* from {D-A} seems straightforward. I don't see any need to regard {D-A} and {D-A}* as orthogonal ensembles.

The ”ET rates”  are calculated by FGR, which is standardly invoked in the theory of ET. When the small parameter is the electronic tunneling matrix element, the FGR expression used here (Eqs. (4-6)) becomes valid (other expressions are obtained in different limits). Importantly, the expression here refers to the strong vibronic coupling limit, and it is not clear to me how it may add anything beyond Marcus theory about the “electronic tunneling”. (They do go beyond Marcus theory in accounting quantum mechanically for the nuclei.)

The manuscript does not address the magnitude of the electronic coupling. However, it goes well beyond Marcus theory by showing how the overall rate of electron transfer depends on the rates of formation and decay of {D-A}* and {DA-}* in addition to the electronic coupling matrix element. The formalism used in the manuscript should hold even if the coupling matrix element is large enough for the Marcus equation to fail.

All in all, it is not clear how the calculations of the ET rates were done, and to what extent the “theoretical results” are fitted to “experimental results”. For example, Table 1 reports allegedly “calculated” numbers. However, some of these numbers require as input the solvent reorganization, where in the text we have: “Herein, solvent reorganization energies are estimated via Eq.s 2 and 4 from the experimental rate constants of the extremely exergonic BIP-BQO or BIP-NQO pairs…” So are the numbers in Table 1 theoretical or experimental? In another place it was stated that k_act is calculated whereas k_deact is taken from experiment. Let alone the possible breakdown of detailed balance by this procedure, are the numbers in Table 2 theoretical or experimental?

Some revision might help to deal with these questions. I don't think they raise any fundamental issues.

The statement: “falling in the inverted region, without requiring a solvent activation step”  is wrong: being in the inverted region does not mean that solvent activation is not needed.

This criticism could be answered easily by deleting the clause "without requiring a solvent activation step", which might have been confusing.
